# Respiratory Syncytial Virus Infections Enhance Cigarette Smoke Induced COPD in Mice

**DOI:** 10.1371/journal.pone.0090567

**Published:** 2014-02-28

**Authors:** Robert F. Foronjy, Abdoulaye J. Dabo, Clifford C. Taggart, Sinead Weldon, Patrick Geraghty

**Affiliations:** 1 St. Luke’s Roosevelt Hospital, Mount Sinai Health System, Division of Pulmonary and Critical Care Medicine, New York, New York, United States of America; 2 Centre for Infection and Immunity, School of Medicine, Dentistry and Biomedical Sciences, Queen’s University Belfast, Belfast, Northern Ireland, United Kingdom; University of Kansas Medical Center, United States of America

## Abstract

Respiratory syncytial viral (RSV) infections are a frequent cause of chronic obstructive pulmonary disease (COPD) exacerbations, which are a major factor in disease progression and mortality. RSV is able to evade antiviral defenses to persist in the lungs of COPD patients. Though RSV infection has been identified in COPD, its contribution to cigarette smoke-induced airway inflammation and lung tissue destruction has not been established. Here we examine the long-term effects of cigarette smoke exposure, in combination with monthly RSV infections, on pulmonary inflammation, protease production and remodeling in mice. RSV exposures enhanced the influx of macrophages, neutrophils and lymphocytes to the airways of cigarette smoke exposed C57BL/6J mice. This infiltration of cells was most pronounced around the vasculature and bronchial airways. By itself, RSV caused significant airspace enlargement and fibrosis in mice and these effects were accentuated with concomitant smoke exposure. Combined stimulation with both smoke and RSV synergistically induced cytokine (IL-1α, IL-17, IFN-γ, KC, IL-13, CXCL9, RANTES, MIF and GM-CSF) and protease (MMP-2, -8, -12, -13, -16 and cathepsins E, S, W and Z) expression. In addition, RSV exposure caused marked apoptosis within the airways of infected mice, which was augmented by cigarette smoke exposure. RSV and smoke exposure also reduced protein phosphatase 2A (PP2A) and protein tyrosine phosphates (PTP1B) expression and activity. This is significant as these phosphatases counter smoke-induced inflammation and protease expression. Together, these findings show for the first time that recurrent RSV infection markedly enhances inflammation, apoptosis and tissue destruction in smoke-exposed mice. Indeed, these results indicate that preventing RSV transmission and infection has the potential to significantly impact on COPD severity and progression.

## Introduction

Chronic obstructive pulmonary disease (COPD) is the third leading cause of death in the US [1] and a foremost cause of morbidity. Indeed, those with the disease have difficulty performing simple daily tasks such as walking, bathing and feeding themselves. The economic costs of COPD are substantial both in terms of healthcare expenditures and lost productivity [2], [3]. Acute exacerbations of COPD, defined as a sudden worsening of COPD symptoms (shortness of breath, quantity and color of phlegm) that typically lasts for several days, are a major contributor to disease morbidity and mortality [4], [5]. Indeed, those with 3 or more exacerbations over a five-year time period had a survival rate of 30% compared to a survival rate of 80% for those without an exacerbation [6]. In the Perception of Exacerbations of Chronic Obstructive Pulmonary Disease (PERCEIVE) survey, 89% of COPD subjects experienced at least one exacerbation in the past year and 21% of these exacerbations resulted in hospital admission [7]. These findings show that exacerbations are common in COPD and impact significantly on the natural history of the disease. Thus, these studies underscore the importance of determining the etiologic factors underlying COPD exacerbations.

Infectious agents, such as viral infections, have been implicated in the pathogenesis of COPD exacerbations [8], [9]. Rhinovirus, influenza and respiratory syncytial virus (RSV) are frequently detected in the respiratory tract of COPD patients [10]. It is estimated that almost everyone has experienced at least one RSV infection as an infant [11] but immunity against RSV infections is uncommon. RSV infections are frequently reported in infants, the elderly and immunocompromised patients but also in healthy adults [12], [13]. RSV is detected in stable COPD patients [14] but whether RSV plays a role in COPD progression is undetermined. RSV readily infects the airway epithelium [11], which could significantly contribute to inflammation and airway disease progression. In fact, infection with RSV is associated with airway inflammation and an accelerated decline in FEV1 in COPD patients [15]. Moreover, the presence of RSV infection worsens outcomes in COPD patients hospitalized for disease exacerbations [16]. Once infected, RSV can persist in the lungs of COPD patients by antagonizing antiviral cytokines, mimicking chemokines, escaping detection through antigenic drift and by entering immune-privileged cells such as pulmonary neurons [17]. Though cigarette smoke impairs antiviral responses that clear RSV [18], [19], the consequence of persistent RSV infection on key disease parameters and outcomes in COPD has yet to be determined and larger clinical studies are need to identify if smoke exposure is a risk factor for increased RSV infections in COPD patients [20].

Few studies have examined the effects of cigarette smoking and viral infections *in vivo* and the subsequent impact on lung morphology [21], [22]. Environmental tobacco smoke has been associated with increased RSV infection and hospitalization in children [23], however little is known about the clinical implications in COPD, a disease that RSV frequently colonizes [15], [24]. Therefore this study aimed to examine how combined RSV infections and smoke exposure would impact on airway inflammation, protease production, apoptosis, lung tissue destruction and phosphatase activity. We hypothesized that dual stimulation could lead to excessive inflammation and an enhanced disease state closer to human COPD. We show that RSV infection enhances cigarette smoke induced immune cell infiltration, cytokine and protease production and airway remodeling. Collectively, these data demonstrate that viral infection alone or in combination with smoke exposure can significantly contribute to airway remodeling and disease progression.

## Methods

### Ethics Statement

All animal procedures performed in this study are in accordance with Institutional Animal Care and Use Committee (IACUC) guidelines, and have been approved by St. Luke’s Roosevelt’s Hospital IACUC at Mount Sinai School of Medicine.

### RSV Culture

Human RSV strain A2 (ATCC, Manassas, VA; #VR-1540) was infected at a multiplicity of 0.1 into Hep2 cells. The virus was allowed to grow for 5 days at 37°C in a 5% CO_2_ atmosphere. The infected Hep2 monolayers were collected and the virus was released by sonication. Cell debris was removed by centrifugation at 2500 g for 5 minutes at 4°C. Virus was collected by centrifuging the supernatant for 2 hours at 22000×g at 4°C. Virus were suspended in culture media and snap frozen and maintained at −80°C. Infectious virus titers were determined on Hep2 cells by performing serial dilutions of the RSV stocks and counting infected cells stained for indirect immunofluorescence with an RSV F-specific monoclonal antibody (Abcam, Cambridge, MA). Additionally, plaque assays were performed as previously described [25] on Hep2 cells using methyl cellulose overlay media (R&D Systems) and staining with 0.5 mg/ml thiazolyl blue tetrazolium bromide (MTT; Sigma Aldrich) solution in PBS for 3 hours at 37°C. Non-infected Hep2 cell cultures were processed in the same manner as RSV infected cells and the resulting sample collection was used as a mock control.

### Cigarette Smoke Exposure and RSV Infection

C57BL/6J mice were purchased from the Jackson Laboratory (Bar Harbor, ME). All mice were maintained in a specific pathogen-free facility at St. Luke’s Roosevelt’s Hospital. 12-week-old mice were used at the initiation point for all experiments and each experimental parameter had 12 animals per group. Mice were anesthetized by intraperitoneal injection of a mixture of ketamine and xylazine. Mice were exposed to cigarette smoke in a chamber (Teague Enterprises, Davis, CA) for four hours a day, 5 days per week at a total particulate matter concentration of 80 mg/m^3^. Smoke exposure was continued for 6 months with RSV dosing or mock administered 2 weeks after initiation of smoke exposures and continued monthly. RSV was intranasally administered at a dose of 1×10^6^ pfu. RSV infected animals were housed separately from non-infected animals. Animals were monitored for discomfort, weight loss and any notable unusual behavior. Body weight and survival were measured every 5 days, for 6 months. Animals were sacrificed 12 hours after the last smoke exposure, which was 10 days after the last RSV or mock administration. The lungs underwent pressure-fixation and morphometric analysis in accordance with our previously published protocol [26] and in accordance with the ATS/ERS issue statement on quantitative assessment of lung structure [27]. Bronchoalveolar lavage fluid (BALF) isolation was performed on the mice. All mouse experiments were carried in strict accordance with institutional protocols.

### Lung Titers and RSV N Copy Number

Lungs of infected mice were excised 10 days post the last RSV infection and homogenized using a mechanical homogenizer (Kinematica, Bohemia, NY, USA). The viral titers in the homogenates were quantified by plaque assay on Hep2 cells. The concentration of RSV N (pg) was determined by PCR based on a standard curve. The following primers were used at 100 pmol each: 5′-TGG GAG AGG TAG CTC CAG AA-3′ and 5′-AGA ATC TGT CCC CTG CTG CTA-3′. Ct was plotted against known RSV standards. A Ct-value of 45 was chosen as the cut-off value for sample infection positive. Results are represented as natural log pico grams (pg).

### Histological Analysis

Fixed tissue was H&E stained for inflammation and fibrosis scoring and mean linear intercept (MLI) determination. Matrix accumulation was assessed on fixed tissue with trichrome evaluations using a commercial available kit (Abcam; ab150686). Histological analysis of H&E stained slides were used to determine perivascular vascular inflammation (PVI) and bronchial inflammation using a modified quantification schema [28], [29]. Briefly, the intensity of perivascular or bronchial inflammation was scored on a scale of 1 to 9. 0, was no inflammation; 1–3, was scant cells but not forming a defined layer; 4–6, one to three layers of cells surrounding the vessel; 7–9, four or greater layers of cells surrounding the vessel or bronchial. Every vascular vessel and bronchus was measured on multiple lung lobes from 3 different depths of sectioned tissue. The validated semiquantitative Ashcroft score was used to score pulmonary fibrosis; scoring from 0 (normal lung) to 8 (total fibrous obliteration of the field) under 100X magnification using trichrome stained sections [30], [31]. Each histology analysis was performed on 12 animals per treatment group.

### Protease and Cytokine Measurements

MMP, cathepsin and cytokine gene expression was performed by qPCR using validated Taqman probes (Life technologies/Applied Biosystems, Carlsbad, CA). RNA was isolated using Qiagen RNeasy kit following tissue homogenizing and cDNA was reverse transcribed using the Applied Biosystems high capacity cDNA kit. qPCR was performed on the Bio-Rad CFX384 real time system. No cDNA template, no reverse transcriptase treated samples and no DNA polymerase controls were examined for each qPCR throughout this study. Exogenous (human targets) and endogenous (Actin) positive controls were also monitored for each assay. All qPCR results are represented as relative quantification (RQ) compared to the mock and room air treated animals and corrected to actin levels. Several MMP and cytokine levels were measured in BALF using a beads assay (EMD Millipore, Billerica, MA) with the BioRad Bio-Plex 200 system (BioRad, Hercules, CA). Cathepsin S activity assays were performed on BALF as previously described [32]. BALF collagenase activity was determined by a colorimetric ninhydrin method, as previously described [33]. Results of enzyme activity are expressed as a relative percentage of activity compared of the mock and room air treated animal group. BALF gelatinase activity was determined by gelatin zymography as described previously [34].

### Intracellular Signaling

Lung tissue protein from mice was homogenized in RIPA buffer, centrifuged at 13,000×*g* for 10 minutes and supernatants collected. Immunoblots were conducted to determine levels of cathepsin E, S, G, K, W, Z (all cathepsin antibodies from Santa Cruz Biotechnology, Paso Robles, CA), MMP-28 (EMD Millipore) and actin (Cell Signaling Technologies, Danvers, MA). Chemiluminescence detection was performed using the Bio-Rad Laboratories Molecular Imager ChemiDoc XRS+ imaging system. Densitometry was performed on each target and represented as a ratio of pixel intensity compared to actin, using Bio-Rad Laboratories Image Lab software (version 4.0, build 16).

### Terminal Deoxynucleotidyl Transferase dUTP Nick End-labeling (TUNEL) Analysis

Apoptosis was determined on paraffin-embedded tissue by the TUNEL in situ cell death detection kit AP (Roche Diagnostics), using the instructions provided by the manufacturer. At least 10 random sections were obtained from each lung from 3 different depths of sectioned tissue. After staining, a minimum of 1000 cells was visually evaluated in each section. The labeled cells were expressed as a percentage of total nuclei.

### Phosphatase Levels

PP2A and PTP1B activities were determined as previously described [35]. qPCR was performed for PTP1B and the A subunit of PP2A using Taqman probes, under the same criteria as described above.

### Statistical Analyses

Data are expressed as means ± S.E.M. We determined statistical significance by Student t-tests (two tailed) using GraphPad Prism Software (Version 5 for Mac OS X). Two-tailed ANOVA repeat measure analysis was employed to determine the changes in animal body weight over time. All data sets are represented as mean +/− standard error.

## Results

### Cigarette Smoke Enhances RSV Infection

**Figure 1:**
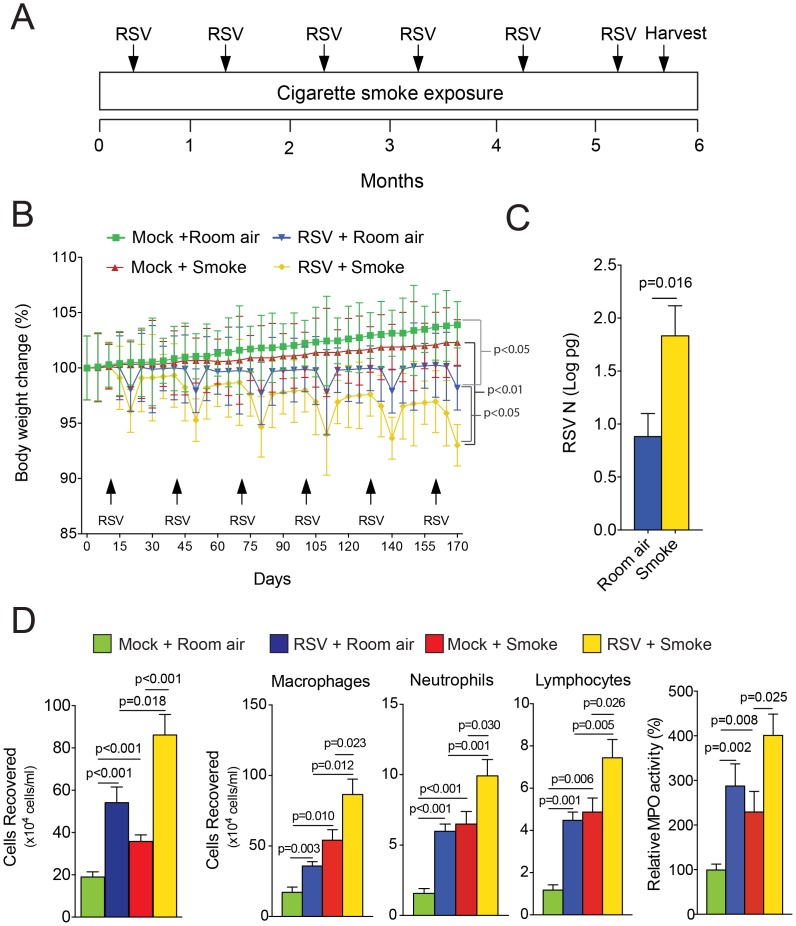
Chronic cigarette smoke exposure enhances RSV pathology in mouse airways. (A) Protocol for administering A2 RSV strain and cigarette smoke to C57BL/6J mice. C57BL/6J mice (n = 12 animals/group) were infected with monthly RSV infections in combination with 6 months of smoke exposure. (B) Changes in body weight of each treatment group of C57BL/6J mice, represented as a percentage of initial weight of mock and room air treated mice at the beginning of this study. p values shown, comparing both groups by 2-way ANOVA. (C) RSV N copy number was determined 10 days post final infection in the lungs of C57BL/6J mice infected with 1×10^6^ pfu of RSV A2 strain and exposed to either room air or cigarette smoke for 6 months. Absolute RSV N concentration was represented as natural log pg. (D) BALF immune cellularity was measured and changes were observed following repeat monthly RSV infections in combination with 6 months of smoke exposure on total immune cell number, macrophages, neutrophils and lymphocytes. Graphs are represented as mean ± S.E.M., where each measurement was performed 3 times on 12 animals/group. p values shown, comparing both treatments connected by a line.

Mice were administered a monthly dose of RSV either in combination with daily cigarette smoke or room air, as depicted in Figure 1A. Animals were sacrificed 10 days after receiving the final dose of RSV, to eliminate a saturation of inflammation persisting from the viral infection. Mice exposed to cigarette smoke gained less weight over the study, which was amplified by repeated RSV infections (Figure 1B). Significant differences in body weight loss were initially found between mock controls and RSV treated mice after 140 days (p<0.05), between smoke and smoke/RSV treated mice after 45 days (p<0.01) and between RSV and smoke/RSV treated mice after 140 days (p<0.05). RSV was still detectable in the lungs of mice 10 days post infection, by qPCR (Figure 1C) but was undetectable by plaque assay (data not shown) as similarly observed by other investigators [25]. Cigarette smoke exposed mice had a higher RSV N copy number than room air exposed mice (Figure 1C). Not surprisingly, exposure to RSV or smoke alone lead to an infiltration of macrophages, neutrophils and lymphocytes into the lung (Figure 1D). Interestingly, smoke exposure synergistically enhanced RSV induced inflammation, with macrophages, neutrophils and lymphocytes.

### RSV Enhances Cigarette Smoke Inflammation and Fibrosis

**Figure 2:**
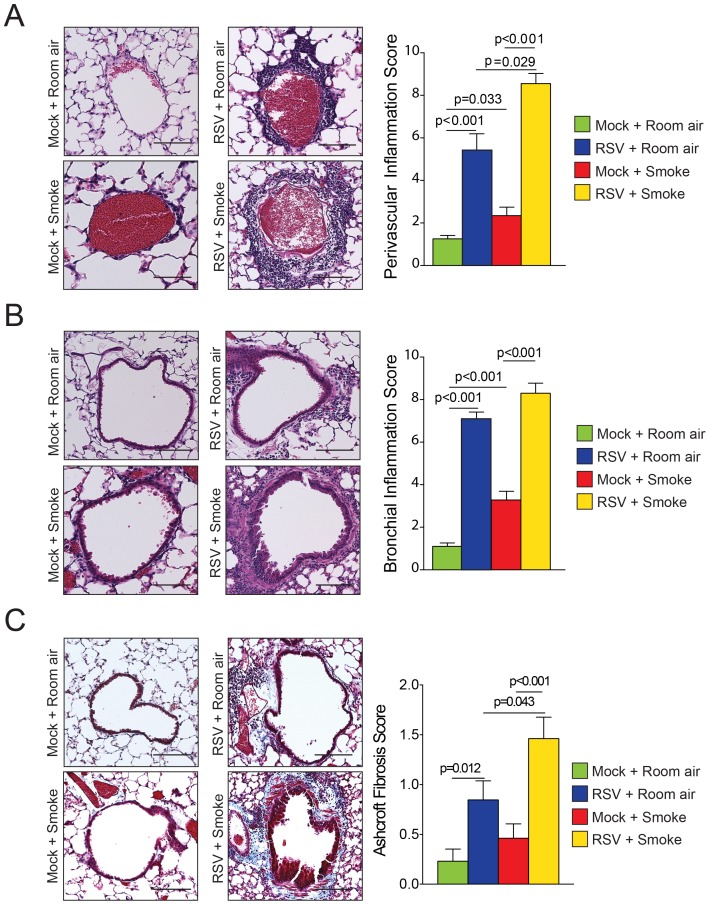
RSV infections enhance cigarette smoke induced airway enlargements and fibrosis. (A) Perivascular and (B) bronchial inflammation was recorded in mice exposed to cigarette smoke and RSV for 6 months and their corresponding controls. (C) Matrix accumulation was assessed with trichrome staining in each mouse group and quantified by the Ashcroft fibrosis score. Representative images of mice lungs from each group are presented here (scale bar = 20 µM; left panels). Fibrosis and inflammation scores were calculated for each treatment group (right panels where n = 12 animals/group). Each graph is represented as mean ± S.E.M. where each measurement was performed on 12 animals/group. p values shown, comparing both treatments connected by a line.

RSV and cigarette smoke-exposed mice exhibited perivascular lymphocytic inflammation, which was additionally augmented by exposure to both RSV and cigarette smoke (Figure 2A). An inflammation score was also performed on the inflammatory cell infiltration around the bronchial airways and the results were comparable to the perivascular inflammation, with RSV enhancing smoke-induced inflammation (Figure 2B). Trichrome staining demonstrated increased airway fibrosis in mice exposed to both cigarette smoke and RSV infections (Figure 2C), with collagen deposition observed around the airways. Ashcroft fibrosis scoring confirmed increased fibrosis in mice exposed to both cigarette smoke and repeat RSV infection (Figure 2C). Therefore, dual stimulation with smoke and RSV infection lead to an exaggerated inflammation and fibrotic airway remodeling response.

### RSV Infections Enhance Cigarette Smoke Induced Airway Remodeling

**Figure 3:**
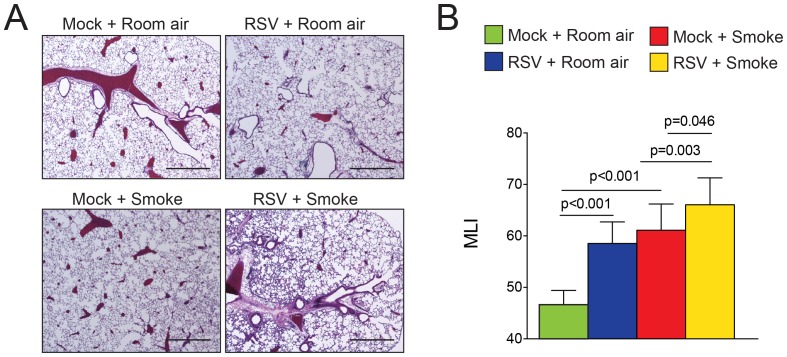
RSV infections enhance cigarette smoke induced airway enlargements. Lung morphology was determined in mice exposed to cigarette smoke and RSV for 6 months and their corresponding controls. (A) Representative images (scale bar = 50 µM) and (B) mean linear intercepts (MLI) of mice lungs from each group are presented here. Graph is represented as mean ± S.E.M., where each measurement was performed on 12 animals/group. p values shown, comparing both treatments connected by a line.

Others have demonstrated that short term smoke exposure in combination with viral infection or poly (i:c) could enhance airway remodeling in animal models [21], [36]. To investigate the long-term impact of combined smoke exposure and multiple viral infections on lung remodeling, we examined airway remodeling by MLI analysis. Not surprisingly, chronic cigarette smoke exposure alone caused a significant increase in alveolar size determined morphometrically by MLI (Figure 3). Interestingly, repeated RSV infection also enhanced airspace enlargement (Figure 3A–B). Combined stimuli of smoke and RSV induced a synergistic enhancement of airway remodeling (Figure 3A–B).

### RSV Infections Alter the Cigarette Smoke Induced Airway Protease Response

**Figure 4:**
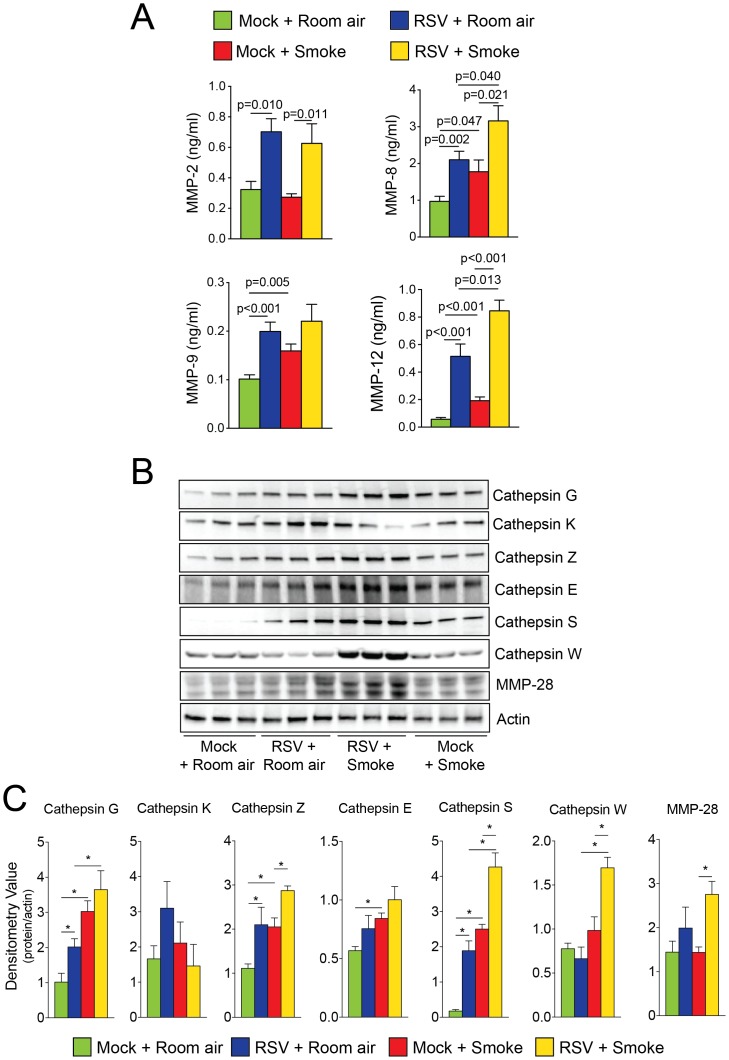
RSV infections enhance cigarette smoke induced airway protease response. (A) BALF MMP (-2, -8, -9 and -12) levels were determined by multiplex analysis in the BALF of mice exposed to cigarette smoke and RSV for 6 months and their corresponding controls. Cathepsin G, K, Z, E, S, W and MMP-28 lung protein expression levels were analyzed by (B) immunoblotting and (C) densitometry. Graphs are represented as mean ± S.E.M., where each measurement was performed 2 times on 12 animals/group. p values shown, comparing both treatments connected by a line.

**Figure 5:**
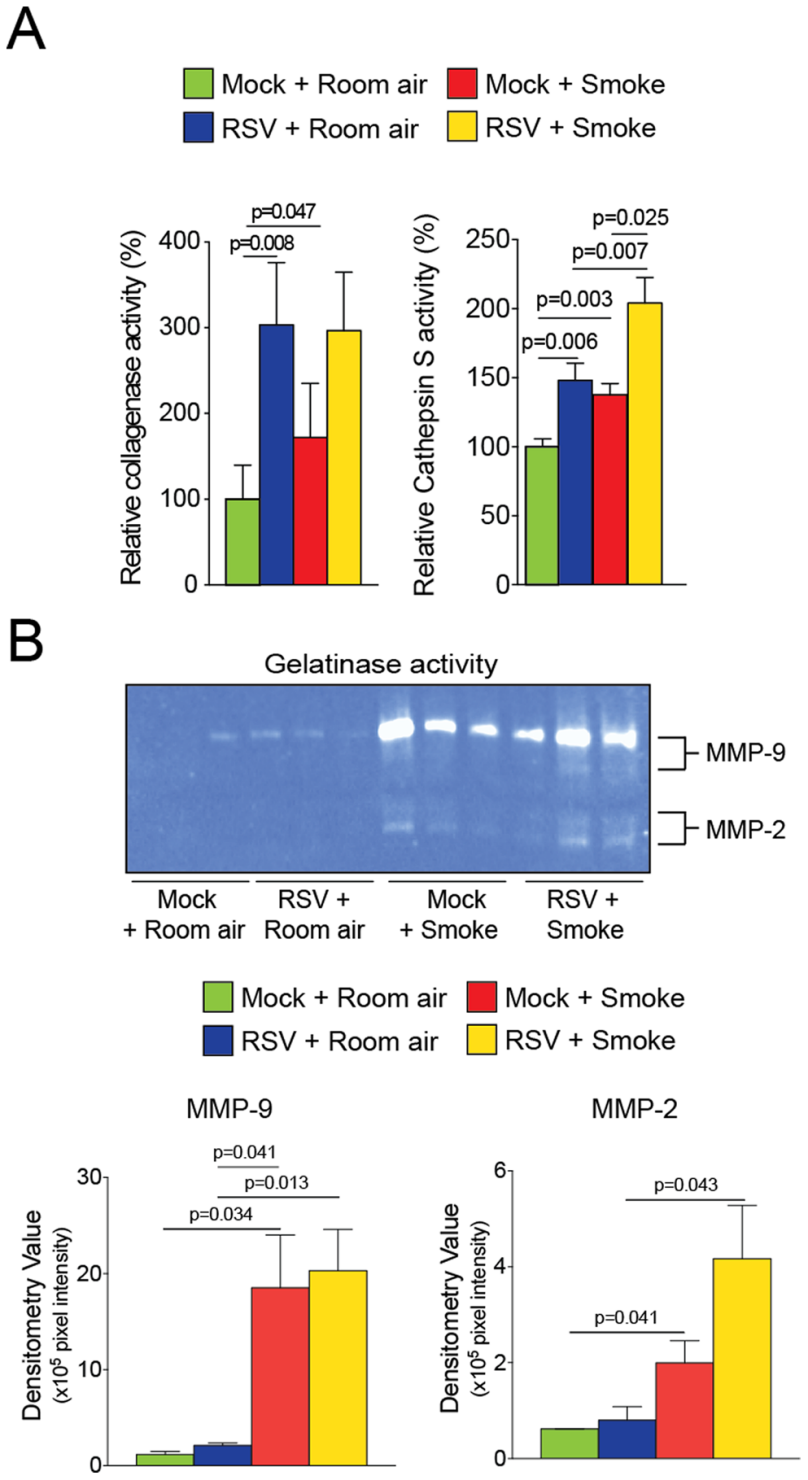
RSV infections enhance cigarette smoke induced cathepsin S activity. BALF protease activity was of mice exposed to cigarette smoke and RSV for 6 months and their corresponding controls. (A) Total BALF collagenase and cathepsin S relative activity was determined. (B) Gelatinase activity was determined in BALF and densitometry was performed for MMP-9 and MMP-2. Graphs are represented as mean ± S.E.M., where each measurement was performed 3 times on 12 animals/group. p values shown, comparing both treatments connected by a line.

Increased protease levels have been frequently observed in human airway diseases [33], [37]. The influence of exposures to viral infection on smoke induced protease expression was investigated by qPCR, multiplex analysis, immunoblots and activity assays from tissue or BALF. C57BL/6J mice infected multiple times with RSV have significant gene expression increases for matrix metalloproteinases (MMP) -8, -9, -12, -13, -14, -16 and cathepsins E, G, M, S, W and Z (Table 1; see Table S1 for gene expression of remaining MMPs and cathepsins). Multiplex assays confirmed increased MMP-2, -8, -9 and -12 in the BALF of RSV infected mice (Figure 4A). RSV exposure also increased tissue protein levels of cathepsin G, S and Z (Figure 4B–C). Smoke exposure induced significant gene expression increases for MMP-8, -9, -12, -14 and cathepsins E, G, M and S (Table 1). Multiplex assays confirmed increased MMP-8, -9 and -12 in the BALF of smoke exposed mice (Figure 4A) and immunoblotting confirmed that smoke induced cathepsin G, Z, E and S levels in tissue (Figure 4C). Combined RSV infection and smoke exposure induced enhanced gene expression of MMP-2, -8, -12, -13, -16, -19, -20, -28 and cathepsins E, S, W and Z (Table 1). Multiplex assays confirmed that smoke enhanced RSV-induced MMP-8 and -12 in the BALF (Figure 4A). Tissue protein levels of cathepsin G, S and W were also synergistically enhanced by smoke (Figure 4B–C). MMP-3 BALF protein levels were also examined to confirm qPCR data of a protease that was unaltered by either stimuli and was confirmed to be unchanged in all mouse groups (Figure S1 and Table S1). Protease activities were also altered within the BALF of mice, with repeat RSV or smoke exposure increasing collagenase, gelatinase and cathepsin S activity in BALF of mice (Figure 5). Repeat RSV exposure enhanced smoke induced cathepsin S activity in BALF of mice but did not appear to enhanced total BALF collagenase activity (Figure 5A). Interestingly, smoke alone increased MMP-9 gelatinase activity levels more so than RSV infection, which varied from the qPCR and multiplex data (Figure 5B). Therefore, RSV infections impacts on smoke associated protease production, which may contribute to the airway remodeling observed in this animal model.

**Table 1:** RSV infections alter cigarette smoke induced airway protease gene expressions.

	Stimuli			
Target	Mock/Room air	Mock/Smoke	RSV/Room air	RSV/Smoke
MMP-2	1.00±0.13	1.21±0.17	1.65±0.30	**4.54±0.41** #
MMP-8	1.00±0.21	2.73±0.38*	3.45±0.83*	**3.82±0.75** #
MMP-9	1.00±0.05	1.93±0.26*	1.81±0.24*	1.23±0.15
MMP-12	1.00±0.13	1.87±0.24*	6.76±1.04*	**7.85±1.56** #
MMP-13	1.00±0.26	0.74±0.11	3.93±0.63*	**4.38±0.60** #
MMP-14	1.00±0.13	1.59±0.04*	1.75±0.16*	1.81±0.16
MMP-16	1.00±0.11	0.72±0.18	1.57±0.20*	**1.88±0.11** #
MMP-19	1.00±0.13	0.78±0.07	1.12±0.20	**1.36±0.17** #
MMP-20	1.00±0.31	0.49±0.16	0.95±0.30	**1.74±0.40** #
MMP-23	1.00±0.19	0.99±0.08	0.47±0.10*	0.75±0.29
MMP-28	1.00±0.23	0.75±0.14	1.22±0.39	**1.89±0.27** #
Cathepsin A	1.00±0.29	1.02±0.04	0.51±0.07	**0.55±0.16** #
Cathepsin E	1.00±0.43	3.99±0.78*	5.82±1.15*	**7.53±1.16** #
Cathepsin F	1.00±0.12	0.80±0.12	0.54±0.02*	0.87±0.19
Cathepsin G	1.00±0.26	2.75±0.52*	3.90±0.34*	4.93±0.76
Cathepsin K	1.00±0.19	1.32±0.25	0.82±0.13	**0.71±0.15** #
Cathepsin L1	1.00±0.06	0.81±0.02*	0.70±0.10*	0.90±0.01
Cathepsin M	1.00±0.16	1.89±0.19*	1.79±0.23*	2.12±0.43
Cathepsin S	1.00±0.11	1.67±0.12*	5.08±0.84*	**7.13±1.42** #
Cathepsin W	1.00±0.03	0.91±0.14	5.35±1.41*	**6.05±0.70** #
Cathepsin Z	1.00±0.16	0.73±0.07	1.62±0.05*	**1.65±0.12** #

Values are represented as mean ± S.E.M., where each measurement was performed 3 times on 12 animals/group. Bold numbers denoted by *represents a p value less than 0.05 compared to mock and room air treated mice.

# denotes a p value less than 0.05 compared to either smoke or RSV treated mice.

### RSV Infections Alter Cigarette Smoke Induced Airway Cytokine Release

Microbial infection in the airways can contribute to disease exacerbations, which have been associated with cytokine and chemokine release from lung-residential cells [21]. C57BL/6J mice infected multiple times with RSV have significant BALF increases for IL-1α, IL-1β, IL-6, IL-10, IL-17, IFN-γ, RANTES and KC (Table 2; see Table S2 for cytokines unaltered by RSV or smoke), determined by multiplex assays. Gene expression analysis also identified increased IL-13, IL-27, CXCL9, CXCL10, CXCL11, G-CSF, IFN-α and IFN-β in the tissue of RSV infected mice (Table 3; see Table S3 for gene expression cytokines unaltered by RSV or smoke). Smoke exposure alone-induced increased BALF protein levels of IL-17, TNF-α and KC (Table 2). Increased gene expressions of IL-13, CXCL9, CXCL10, CXCL11, RANTES, IFN-α, IFN-β and IFN-γ were observed after 6 months of smoke exposure (Table 3). Combined smoke and RSV infection lead to an enhanced induction of IL-1α, IL-17, IFN-γ and KC in the BALF and IL-13, IL-27, CXCL9, RANTES, MIF and GM-CSF by qPCR (Table 2 and Table 3). Therefore, combined exposure to cigarette smoke and RSV can synergistically enhance certain immune responses. Equally, trend decreases in several BALF cytokines were observed following dual stimulation of smoke and RSV infection, such as IL-2, IL-3, IL-5, IL-7, IL-9, IL-10, IL-12p70, IL-13, TNF-α, VEGF, MIP1α, and MIP1β (Tables 2 and S2). Altered cytokine levels were probably not dependent on AP-1 or NF-κB activation, as dual exposure did not enhance activity of either transcription factor (Figure S2). However, additional studies are required to fully confirm this observation.

**Table 2:** RSV infections alter cigarette smoke induced airway cytokine release.

Cytokine (pg/ml)	Mock/Room air	RSV/Room air	Mock/Smoke	RSV/Smoke
IL-1α	2.07±0.36	30.11±9.63*	1.11±0.25	**74.35±8.62** #
IL-1β	59.09±6.20	78.75±5.32*	55.73±3.76	75.60±8.40
IL-3	27.91±0.83	22.77±0.98*	24.82±0.91*	21.76±0.80
IL-5	4.68±0.09	4.07±0.20*	4.16±0.13*	4.37±0.36
IL-6	3.78±0.44	9.631±3.81*	4.568±0.38	9.147±2.75
IL-10	61.43±9.92	76.45±12.60*	35.49±2.98*	59.78±18.34
IL-13	48.41±2.44	29.61±3.50*	40.23±1.77	**33.59±2.65** #
IL-17	43.66±5.06	64.17±6.72*	81.62±10.70*	**122.40±7.52** #
IFN-γ	12.15±0.72	35.47±11.26*	15.49±1.30	**80.15±12.02** #
RANTES	12.41±0.84	107.30±11.7*	12.38±1.03	129.1±11.73
TNF-α	524.90±41.34	438.80±55.48	609.10±29.10*	**508.4±58.42** #
MIP-2	10.51±0.27	9.10±0.18*	9.31±0.29*	9.53±0.34
GM-CSF	39.72±2.99	30.49±1.30*	35.00±2.00	33.88±2.64
CXCL1/KC	6.79±1.34	**18.94±2.13**	**12.83±1.85**	**21.59±1.82**

Values are represented as mean ± S.E.M., where each measurement was performed 3 times on 12 animals/group. Bold numbers denoted by *represents a p value less than 0.05 compared to mock and room air treated mice.

# denotes a p value less than 0.05 compared to either smoke or RSV treated mice.

**Table 3:** RSV infections alter cigarette smoke induced airway cytokine gene expressions.

	Stimuli			
Target	Mock/Room air	RSV/Room air	Mock/Smoke	RSV/Smoke
IL-1β	1.00±0.08	2.92±0.30*	2.04±0.18	2.890±0.43
IL-6	1.00±0.25	3.08±0.64*	2.00±0.14	2.43±0.09
IL-10	1.00±0.37	3.42±0.68*	2.12±0.63	2.00±0.34
IL-13	1.00±0.43	6.57±1.55*	14.01±2.71*	**17.17±3.70** #
IL-17	1.00±0.28	3.61±0.66*	2.69±0.40*	**6.69±1.45** #
IL-27	1.00±0.21	2.66±0.53*	1.41±0.21	**3.95±1.42** #
CXCL1/KC	1.00±0.14	1.85±0.24*	2.61±0.23*	**3.42±0.31** #
CXCL9	1.00±0.29	14.47±1.01*	5.69±1.43*	**17.75±2.35** #
CXCL10	1.00±0.42	4.88±0.62*	2.65±0.24*	2.86±0.22
CXCL11	1.00±0.09	6.64±1.35*	3.47±0.88*	4.25±1.22
RANTES/CCL5	1.00±0.19	13.44±2.59*	2.99±1.02*	**19.04±5.40** #
MIF	1.00±0.14	0.49±0.04*	0.87±0.08	**1.43±0.05** #
GM-CSF	1.00±0.11	1.60±0.20	1.59±0.30	**2.44±0.36** #
G-CSF	1.00±0.40	2.11±0.65*	1.20±0.53	1.07±0.19
IFN-α	1.00±0.25	3.81±0.91*	4.15±1.05*	2.87±1.17
IFN-β	1.00±0.15	4.71±0.83*	2.33±0.38*	2.88±0.65
IFN-γ	1.05±0.30	14.61±2.23*	2.64±0.62*	**20.10±3.61** #

Values are represented as mean ± S.E.M., where each measurement was performed 3 times on 12 animals/group. Bold numbers denoted by *represents a p value less than 0.05 compared to mock and room air treated mice.

# denotes a p value less than 0.05 compared to either smoke or RSV treated mice.

### RSV Infections Enhance Cigarette Smoke Induced Airway Cell Death

**Figure 6:**
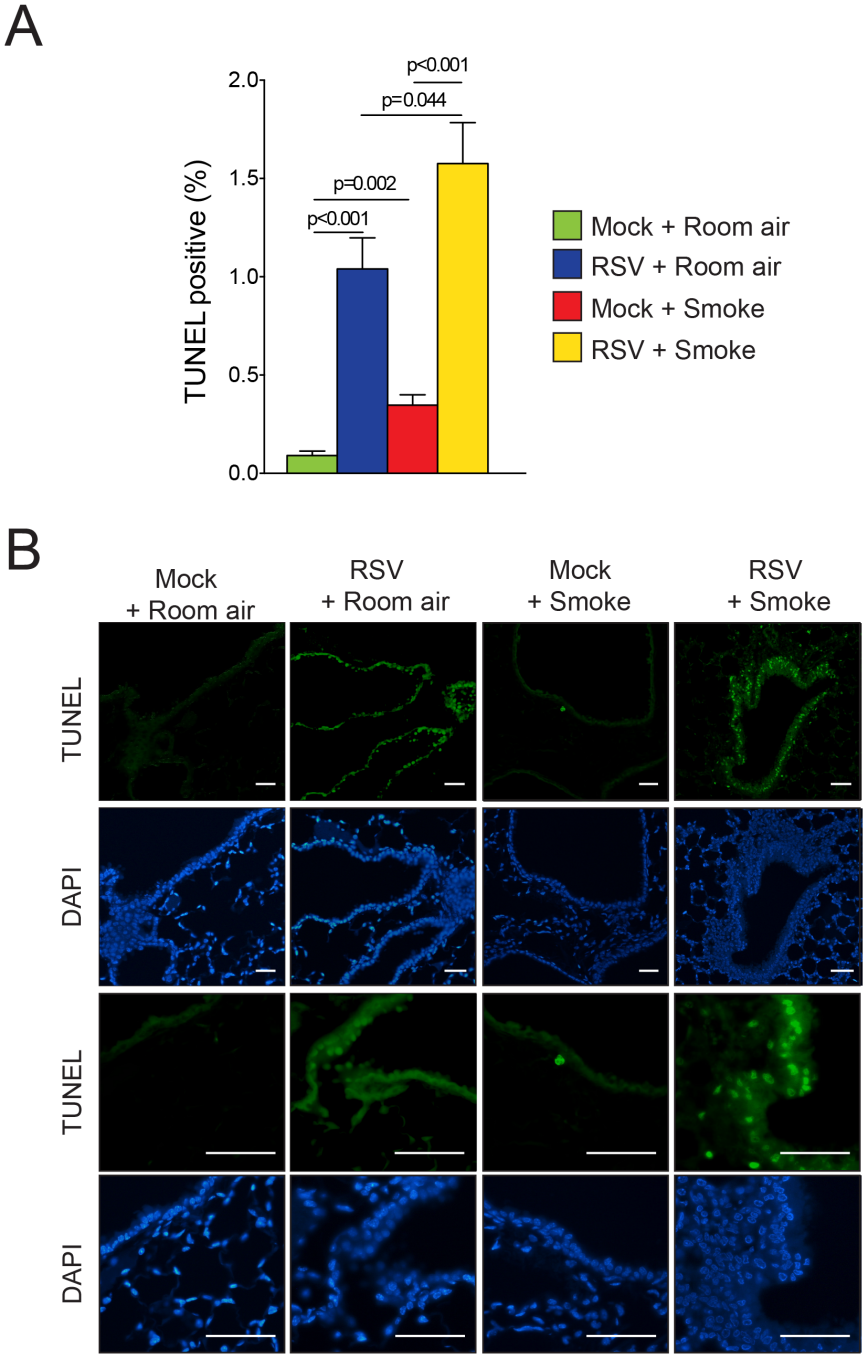
RSV infections enhance cigarette smoke induced airway cell death. (A) TUNEL analysis was performed on lung tissue from mice exposed to cigarette smoke and RSV for 6 months and their corresponding controls. Graph represented as mean ± S.E.M., where each measurement was performed on 12 animals/group. p values shown, comparing both treatments connected by a line. (B) Representative images of TUNEL staining of mice lungs from each group are presented here (scale bar = 50 µM).

Studies were undertaken to evaluate the impact of RSV and smoke exposure on cell death responses in the mouse lung. In cell culture, cigarette smoke causes necrosis rather than virus-induced apoptosis [38]. Our *in vivo* analysis demonstrated that administration of either smoke or RSV to mice caused significant increases in the number of cells that were undergoing apoptosis (Figure 6). RSV caused significant increase in the number of TUNEL positive cells. Smoke alone induced a modest induction of TUNEL positive cells within the lung. However, the combined exposure to smoke and RSV induced the most impressive changes with the detection of many TUNEL positive cells (Figure 6). An increase in the number of TUNEL-positive cells was seen in the bronchial airways of smoke exposed mice that were treated with RSV (Figure 6B).

### RSV Infection Subdued PTP1B and PP2A Expressions and Activities

**Figure 7:**
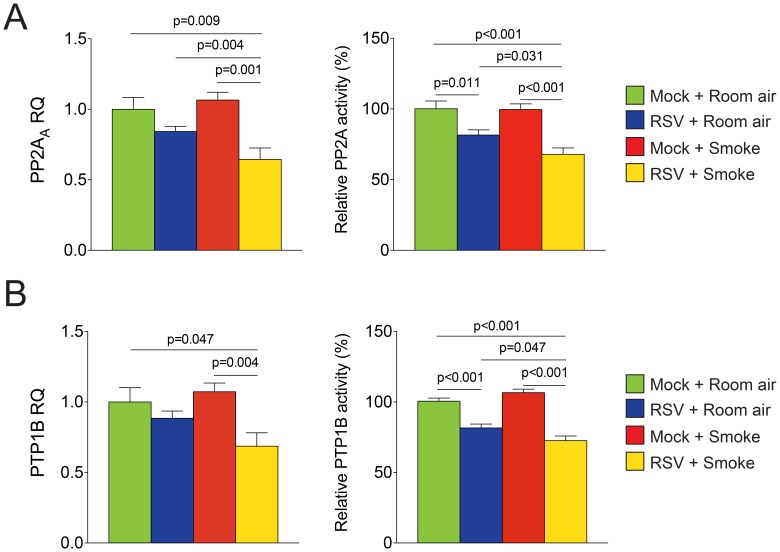
Smoke enhances RSV inhibition of PTP1B and PP2A activities. Lung tissue from C57BL/6J mice was examined for gene expression and phosphatase activity for (A) PP2A and (B) PTP1B. Graphs are represented as mean ± S.E.M., where each measurement was performed on 12 animals/group. p values shown, comparing both treatments connected by a line.

Chronic cigarette smoke exposure subdues the PTP1B and PP2A anti-inflammation response [35], [39] and viruses can prevent PP2A activity [40]. Therefore, we investigated whether dual exposure to smoke and RSV infections could enhance inflammation by preventing the inductions of airway PTP1B and PP2A. As observed before [35], [39], chronic smoke exposure did not induce PTP1B and PP2A activity (Figure 7). RSV inhibited PP2A and PTP1B activities, which was further enhanced by smoke exposure (Figure 7). Exposure to both smoke and RSV significantly reduced gene expression of both phosphatases (Figure 7).

## Discussion

An exacerbation is a seminal event in the life of a COPD patient that frequently marks the transition from relative stability to a more rapid decline in lung function. Given the importance of exacerbations in the severity and progression of the disease, it is critical to establish the mechanisms by which an exacerbation leads to a decline in lung function. It has long been observed that RSV infections are frequently detected in the lungs of COPD patients during an exacerbation [16]. Moreover, RSV is known to persist in the lung even after an exacerbation has appeared to resolve [17]. What this study shows is that the presence of RSV infection exacerbates the underlying inflammatory, proteolytic and apoptotic responses triggered by cigarette smoke exposure in the lung. Indeed, RSV exposure significantly enhanced lung tissue destruction in smoke exposed mice. This study is also the first study to examine the impact of repeat viral infections in animals actively exposed to smoke throughout the study and suggests that inhibition of phosphatase activities may contribute to the inflammation observed in COPD viral exacerbations. Furthermore, these results indicate the specific strategies aimed at preventing and treating RSV infection would have a significant impact on COPD development.

RSV exposure exacerbated the protease/anti-protease imbalance in the lungs of the smoke-exposed mice. This finding was not altogether surprising as RSV induces MMP-3 and -10 expression in nasal epithelial cells [41], [42], MMP-9 expression in human bronchial epithelial cells [43], [44] and MMP-9 and -2 in a BALB/c mouse model [45]. However, the diversity of proteases that were regulated by RSV was impressive and this likely has a wide-ranging biological impact on the lung. Certainly, the role of proteases in emphysema is well established [46], [47] and RSV induced emphysematous changes in mice that were further exacerbated by cigarette smoke exposure. It is conceivable that the proteolytic response may mediate the clearance of RSV infections from the lung. Indeed, collagen and elastin peptides exert chemotactic effects that draw in inflammatory cells that are needed for the elimination of viral infections in the lung [48]. However, it is also possible that proteases may delay viral clearance by degrading proteins that bind RSV and eliminate it from the lung. In fact, proteases degrade surfactant protein A [49], which clears RSV from the lung [50], as well as secretory leukoprotease inhibitor (SLPI), elafin and SerpinB1, which have been reported to exert antiviral effects [51], [52]. Whether all of the proteases identified in this study are present in human viral exacerbations or contribute to disease progression or viral clearance is yet to be determined but the role of each protease in COPD progression represents many potential future topics of interest. The mechanism by which RSV enhances smoke induced proteases also represents an important area for future investigation. Smoke and RSV have several common targets that could impact on protease production, such as TLR4 [53], [54], TLR9 [53], RIG-I [21]. We have previously observed increased protease induction in the absence of PP2A [39] and PTP1B [35] expression. Loss of phosphatase responses could play a major part on the proteases expressed in this study. Equally, phosphatases can regulate cytokine production, which may also contribute to the protease production observed in this study.

The induction of cytokines is necessary to clear viral infections like RSV that contribute to lung injury and disease progression. Many of the cytokines induced by RSV exposure in our model have also been linked with apoptosis, protease expression, mucus metaplasia and lung tissue destruction in COPD [55], [56]. Virus-associated exacerbations have greater loss in lung function and increased CCL5, CXCL10, and CXCL11 [57], which were also observed in our mouse model. COPD patients have increased IFN-γ in BALF fluid [32], possibly from smoke induced CD8+ T cells that are a key regulator of the inflammation response in COPD [58]. IFN-γ primes cytotoxic T cell responses against RSV infection that are important for eliminating the virus from the lung. However, persistent or repeated viral infections, as occurred in our model, leads to chronic upregulation of IFN-γ in the lung, which induces protease expression, apoptosis and emphysema in mice [55] and is associated with disease severity in humans. Loss of PTP1B expression leads to increased smoke induced IFN-γ and IL-17 in the lungs of mice [35]. IL-17, which was induced in our model, modulates airway hyperreactivity and emphysema formation in mice [59]; however, the expression of IL-17 is required for the clearance of bacterial and viral infections in the lung. Indeed, there is some data to suggest that deficient cytokine responses could impair viral clearance from the lung [60], which based on our findings would exacerbate inflammatory and emphysematous changes in the lung. NF-κB and interferon stimulatory response element (ISRE) have been associated with smoke enhanced RSV stimulated cytokines [61]. We do not see a synergistic enhancement of NF-κB activation but we specifically examined animals at the end of the infection. Perhaps smoke may alter NF-κB earlier during the infection. This and the role of ISREs in viral and smoke responses will be an area of future investigation.

Large increases in TUNEL positive epithelial cells were detected in our RSV infected mice and this increase was further enhanced by smoke exposure. This is significant as there have been conflicting reports about the role of RSV in airway epithelial cell death and survival. One group showed that RSV inhibits apoptosis and prolongs survival by downregulating p53 or stimulating epidermal growth factor receptor (EGFR) and phosphatidylinositol 3-kinase (PI3K) signaling in lung epithelial cells [62], [63], [64]. On the other hand, others have reported that RSV sensitizes the epithelium to apoptosis by strongly upregulating the expression of tumor necrosis factor-related apoptosis-inducing ligand (TRAIL) [65] or CD95 (fas) [66], [67]. Emphysematous airways are extremely sensitivity to TRAIL-mediated apoptosis [68], which may account for the increased apoptosis observed in our study following smoke and RSV exposures. TRAIL receptors are overexpressed in human emphysema patients [69] and their expression is sensitive to oxidative stress such as H_2_O_2_ stimulation. RSV infections coincide with increased soluble TRAIL levels in the BALF of respiratory failure patients [70]. Equally, RSV has been shown to reduce NRF2 in animal models [71]. NRF2 regulates numerous cell survival genes [72] and anti-oxidants [71] that are key players in mediating inflammation [35], [73]. PTP1B and PP2A activities are sensitive to oxidation [35] and inhibition of PP2A increases sensitivity to TRAIL signaling [74]. It is interesting to note that the intense apoptotic responses in the airways in this study were accompanied by significant airway inflammation and fibrosis. Thus, determining how epithelial cell death leads to airway fibrosis would shed important insights in the pathophysiologic changes that occur in this disease.

Little clinical data is available on RSV susceptibility and inflammatory markers in COPD patients. However other viruses can give us a potential depiction of the human airway responses to RSV. Experimental rhinovirus studies in humans confirm that viral load correlates strongly with inflammatory markers [60]. BALF cells from COPD patients infected *ex vivo* with rhinovirus have deficient IFN-β induction, which coincides with reduced IFN-stimulated gene CXCL10 [60]. However, others have reported enhanced inflammation in tracheobronchial cells from COPD patients compared to healthy donor cells [75]. Our *in vivo* model observed enhanced inflammation from BALF and lung tissue after RSV infection. Future clinical studies will be required to determine how RSV infection impacts on the inflammatory responses in COPD.

The role of RSV in the progression of COPD has been debated as the virus is detected in up to a third of stable COPD subjects [17]. However, our findings affirm that RSV is not merely a colonizer of the lung but rather a key etiologic factor driving cytokine, protease and apoptotic responses that lead to air space enlargement and airway fibrosis. In fact, RSV exposure by itself induced biological and structural changes that were at least as severe as cigarette smoke exposure. Whether smoke exposure is enhancing RSV inflammation synergistically or accumulatively still remains to be determined but our data suggests that synergistic inhibition of phosphatase activities may be playing a major role in COPD exacerbations. These results provide a clear rationale for developing better RSV prevention and treatment strategies as a means of countering the progression and severity of this disease. This data indicates that the detecting RSV may help identify those subjects who are likely to experience a clinical deterioration over time.

## Supporting Information

Figure S1 RSV infections and cigarette smoke had no impact on MMP-3 BALF levels. BALF MMP-3 levels were determined by multiplex analysis in the BALF of mice exposed to cigarette smoke and RSV for 6 months and their corresponding controls. Graph is represented as mean ± S.E.M., where each measurement was performed 2 times on 12 animals/group.
(TIF)

Figure S2 AP-1 and NF-κB activities following RSV and smoke stimuli. Transcription factor (AP-1 and NF-κB) activation was examined in the lungs of mice exposed to cigarette smoke and RSV for 6 months and their corresponding controls. Graphs are represented as mean ± S.E.M., where each measurement was performed 2 times on 12 animals/group. p values shown, comparing both treatments connected by a line.
(TIF)

Table S1 Protease gene responses in airways to RSV infections and cigarette smoke exposure.
(PDF)

Table S2 Cytokine release in airways to RSV infections and cigarette smoke exposure.
(PDF)

Table S3 Cytokine gene responses in airways to RSV infections and cigarette smoke exposure.
(PDF)

Methods S1 NFB and AP1 activation.
(PDF)
