# Supplementary figures and images for: Respiratory Syncytial Virus Infections Enhance Cigarette Smoke Induced COPD in Mice
Source: PLoS One. 2014 Feb 28;9(2):e90567. doi: 10.1371/journal.pone.0090567 (PMC3938768; doi:10.1371/journal.pone.0090567)

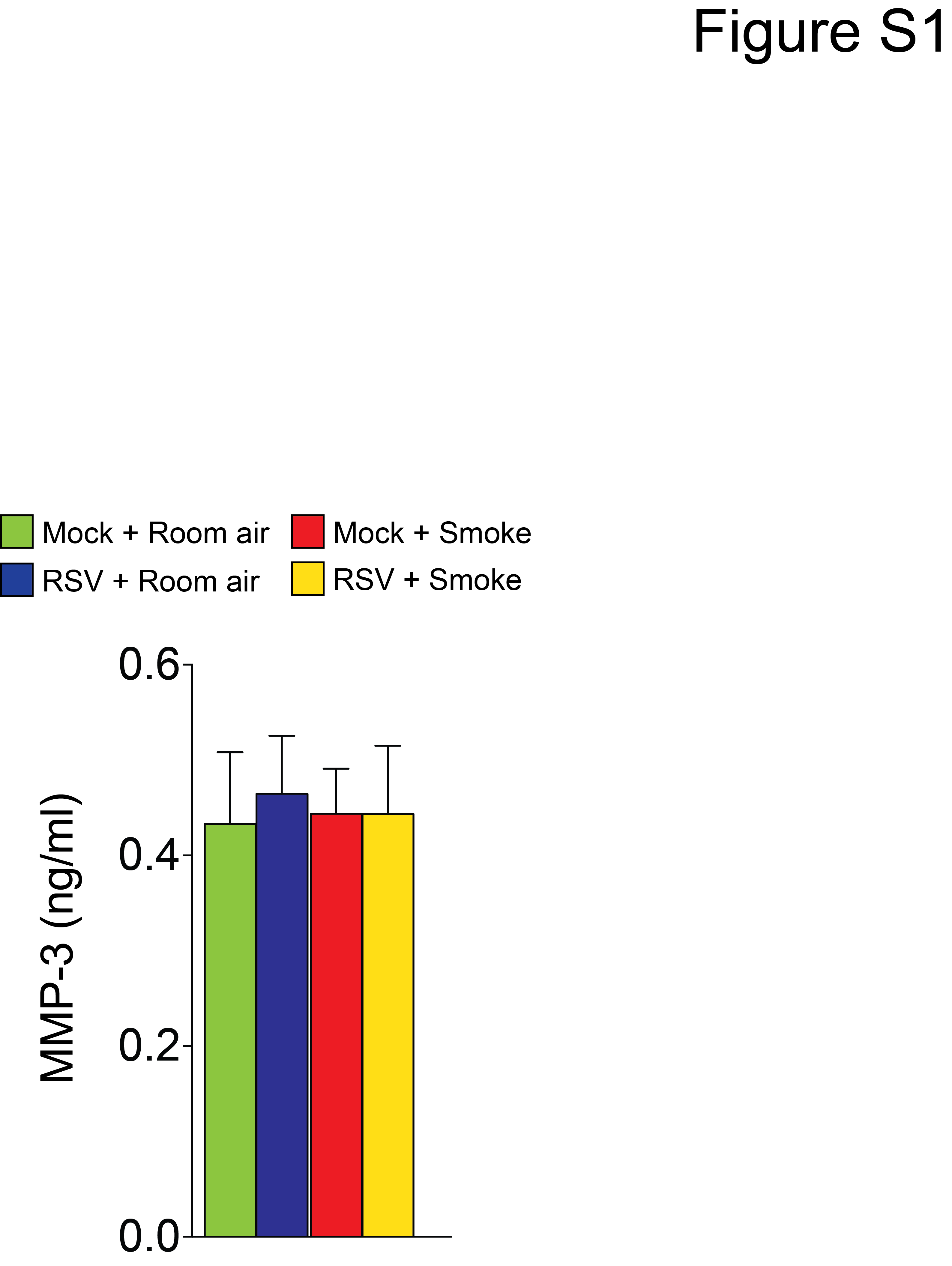

Supplement: Figure S1 — RSV infections and cigarette smoke had no impact on MMP-3 BALF levels. BALF MMP-3 levels were determined by multiplex analysis in the BALF of mice exposed to cigarette smoke and RSV for 6 months and their corresponding controls. Graph is represented as mean ± S.E.M., where each measurement was performed 2 times on 12 animals/group. (TIF) [file pone.0090567.s001.tif]

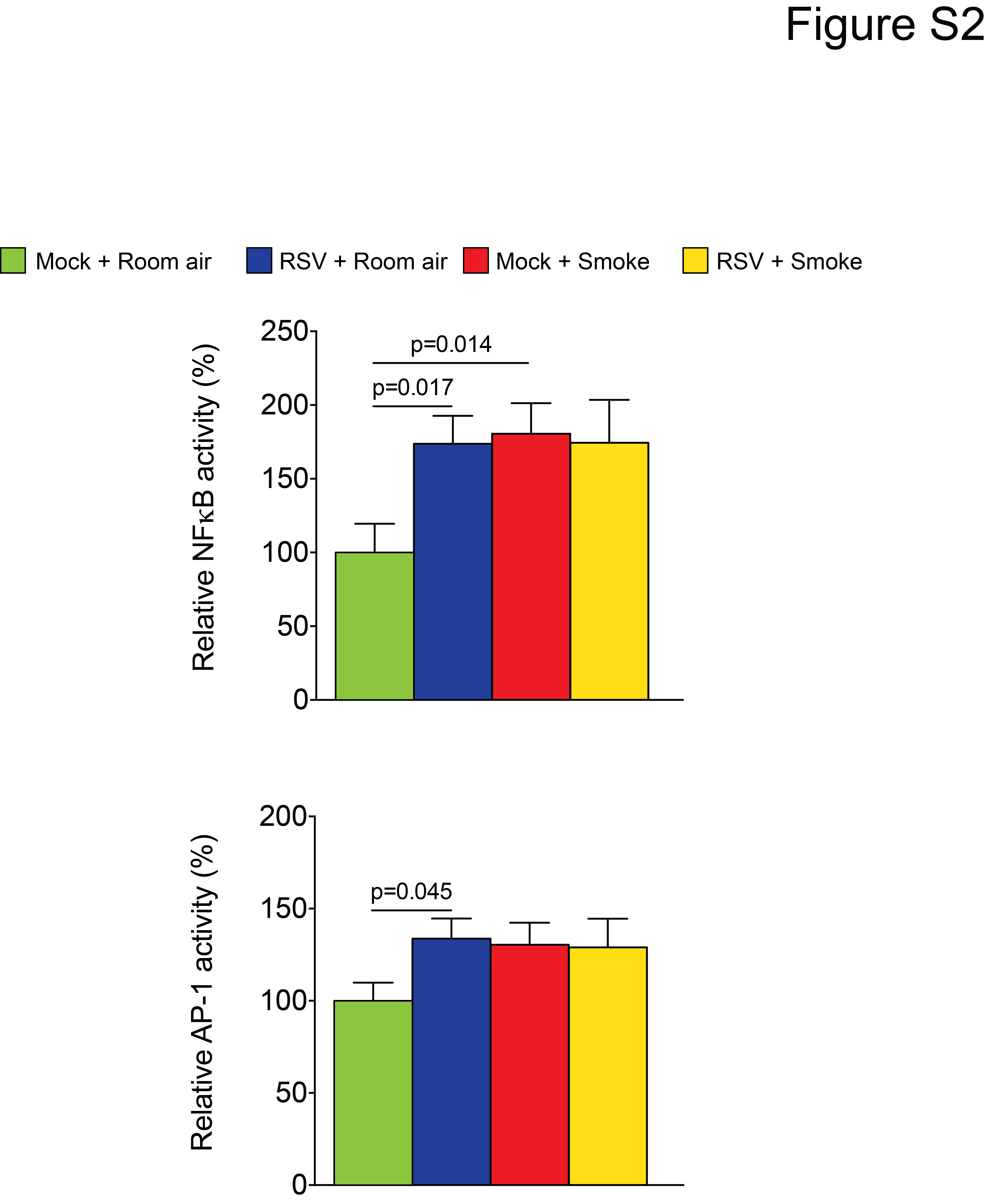

Supplement: Figure S2 — AP-1 and NF-κB activities following RSV and smoke stimuli. Transcription factor (AP-1 and NF-κB) activation was examined in the lungs of mice exposed to cigarette smoke and RSV for 6 months and their corresponding controls. Graphs are represented as mean ± S.E.M., where each measurement was performed 2 times on 12 animals/group. p values shown, comparing both treatments connected by a line. (TIF) [file pone.0090567.s002.tif]
