# Supplementary material for: Respiratory Syncytial Virus Infections Enhance Cigarette Smoke Induced COPD in Mice
Source: PLoS One. 2014 Feb 28;9(2):e90567. doi: 10.1371/journal.pone.0090567 (PMC3938768; doi:10.1371/journal.pone.0090567)
Supplement: Table S1 — Protease gene responses in airways to RSV infections and cigarette smoke exposure. (PDF) [file pone.0090567.s003.pdf]

Table S1. Protease gene responses in airways to RSV infections and cigarette smoke exposure.

	Stimuli			
**Target**	**Mock/Room air**	**Mock/Smoke**	**RSV/Room air**	**RSV/Smoke**
MMP-3	1.00±0.34	1.23±0.14	0.79±0.23	0.79±0.10
MMP-7	1.00±0.51	1.72±0.59	3.28±1.18	2.55±0.56
MMP-10	1.00±0.20	0.61±0.17	1.24±0.37	0.84±0.15
MMP-11	1.00±0.18	0.92±0.11	1.18±0.21	1.21±0.34
MMP-15	1.00±0.29	0.75±0.06	0.53±0.04	0.60±0.13
MMP-17	1.00±0.18	1.26±0.24	0.76±0.10	0.88±0.15
MMP-21	1.00±0.17	0.78±0.14	0.59±0.20	1.24±0.41
MMP-24	1.00±0.24	0.62±0.20	0.62±0.12	0.76±0.25
MMP-25	1.00±0.12	1.09±0.41	1.24±0.20	1.45±0.12
MMP-27	1.00±0.23	0.87±0.24	0.81±0.21	1.24±0.23
Cathepsin B	1.00±0.11	1.14±0.11	0.81±0.05	0.84±0.16
Cathepsin C	1.00±0.07	1.13±0.08	1.16±0.12	1.43±0.18
Cathepsin D	1.00±0.13	1.30±0.15	1.32±0.13	1.67±0.30
Cathepsin H	1.00±0.18	1.12±0.24	0.99±0.08	1.18±0.25
Cathepsin O	1.00±0.07	1.17±0.11	1.06±0.11	1.25±0.06

Values are represented as mean ± S.E.M., where each measurement was performed 3 times on 12 animals/group.
