# Supplementary material for: Respiratory Syncytial Virus Infections Enhance Cigarette Smoke Induced COPD in Mice
Source: PLoS One. 2014 Feb 28;9(2):e90567. doi: 10.1371/journal.pone.0090567 (PMC3938768; doi:10.1371/journal.pone.0090567)
Supplement: Table S2 — Cytokine release in airways to RSV infections and cigarette smoke exposure. (PDF) [file pone.0090567.s004.pdf]

Table S2. Cytokine release in airways to RSV infections and cigarette smoke exposure.

Cytokine (pg/ml)	Mock/Room air	RSV/Room air	Mock/Smoke	RSV/Smoke
IL-2	7.54±1.52	6.10±0.46	6.40±0.53	5.12±0.41
IL-4	0.14±0.01	0.15±0.01	0.14±0.02	0.14±0.02
IL-7	0.43±0.09	0.36±0.12	0.30±0.10	0.30±0.15
IL-9	243.10±33.95	213.30±9.07	203.80±6.19	192.70±5.65
IL-12p40	8.47±0.57	7.93±0.33	7.91±0.34	8.29±0.52
IL-12p70	12.15±2.41	8.17±1.24	9.92±1.80	8.78±1.45
IL-15	8.44±2.31	6.55±1.78	4.10±1.14	3.38±0.77
MCP-1	64.57±2.12	75.14±8.23	71.77±3.60	86.99±16.70
VEGF	2.02±0.33	1.75±0.41	1.22±0.24	1.69±0.25
MIP-1α	104.10±3.12	98.13±1.20	98.99±1.11	96.19±1.23
MIP-1β	67.25±2.73	60.74±3.39	61.95±1.80	60.71±2.31
Eotaxin	5.63±0.41	5.50±0.17	5.72±0.17	5.89±0.32
M-CSF	20.76±0.55	21.74±1.4	20.24±1.00	19.52±0.80

Values are represented as mean ± S.E.M., where each measurement was performed 3 times on 12 animals/group.
