# Supplementary material for: Respiratory Syncytial Virus Infections Enhance Cigarette Smoke Induced COPD in Mice
Source: PLoS One. 2014 Feb 28;9(2):e90567. doi: 10.1371/journal.pone.0090567 (PMC3938768; doi:10.1371/journal.pone.0090567)
Supplement: Table S3 — Cytokine gene responses in airways to RSV infections and cigarette smoke exposure. (PDF) [file pone.0090567.s005.pdf]

Table S3. Cytokine gene responses in airways to RSV infections and cigarette smoke exposure.

	Stimuli			
**Target**	**Mock/Room air**	**RSV/Room air**	**Mock/Smoke**	**RSV/Smoke**
IL-4	1.00±0.13	2.37±0.98	2.10±0.89	2.95±1.27
IL-12	1.00±0.15	1.64±0.11	1.15±0.20	1.97±0.32
IL-18	1.00±0.18	1.32±0.16	1.47±0.20	0.97±0.12
IL-22	1.00±0.24	1.03±0.49	1.14±0.38	0.81±0.37
IL-23	1.00±0.30	0.94±0.24	1.66±0.19	1.84±0.19
TGF-β	1.00±0.21	1.47±0.22	1.06±0.19	1.46±0.30
VEGF	1.00±0.35	1.27±0.19	0.97±0.21	1.53±0.51

Values are represented as mean ± S.E.M., where each measurement was performed 3 times on 12 animals/group.
