# Supplementary material for: Respiratory Syncytial Virus Infections Enhance Cigarette Smoke Induced COPD in Mice
Source: PLoS One. 2014 Feb 28;9(2):e90567. doi: 10.1371/journal.pone.0090567 (PMC3938768; doi:10.1371/journal.pone.0090567)
Supplement: Methods S1 — NFB and AP1 activation. (PDF) [file pone.0090567.s006.pdf]

SUPPLEMENTAL METHODS

NFB and AP1 activation

NF-κB and AP-1 activation were measured on the nuclear protein extracts using specific activation assays from Active Motif (46096 and 40096; Active Motif, Carlsbad, CA, USA). Results of transcription factors activity are represented as relative activity as a percentage compared to the vehicle treated group.
